# Aspects épidemiocliniques et évolutifs chez 157 cas de leishmaniose cutanée au Maroc

**DOI:** 10.11604/pamj.2014.17.272.3268

**Published:** 2014-04-14

**Authors:** Naoufal Hjira, Rachid Frikh, Tarik Marcil, Hanane Lamsyah, Siham Oumakhir, Noureddine Baba, Mohammed Boui

**Affiliations:** 1 Service de Dermatologie, Hôpital Militaire d'Instruction Mohammed V Rabat, Maroc

**Keywords:** Leishmaniose cutanée, leishmania tropica, leishmania major, Glucantime, Maroc, cutaneous leishmaniasis, leishmania tropica, leishmania major, Glucantime, Morocco

## Abstract

Connue au Maroc depuis la fin du XIX siècle, la leishmaniose cutanée (LC) constitue un problème de santé publique dans notre pays. Le but de notre travail est de décrire le profil épidémioclinique et l’évolution post thérapeutique chez les patients ayant une leishmaniose cutanée dans notre contexte. Nous avons effectué une étude rétrospective, basée sur l'exploitation des dossiers de malades ayant présenté une leishmaniose cutanée confirmée entre janvier 2003 et décembre 2012. Nous avons colligés 157 cas de leishmaniose cutanée. L’âge moyen des patients était de 34.5 ans avec des extrêmes de 6 ans à 63 ans. Le sex-ratio était de 2.34 H/F. La durée d’évolution moyenne des lésions était de 3,6 mois avec des extrêmes de 2 semaines à 10 mois. Les lésions étaient uniques dans 29.5% des cas. Les lésions siégeaient sur membres dans 63%. La forme ulcèro- croûteuse touchait plus de 48%. Le Glucantime était utilisé dans 29.3% des cas, l'azote liquide était utilisé chez 111 autres. L’évolution post-thérapeutique était favorable avec disparition quasi-complète des lésions dans un délai variant de 6 à 10 semaines, au prix de cicatrices inesthétiques chez 14 patients. La leishmaniose cutanée continue à poser un vrai problème de santé publique dans notre pays. L’émergence de formes sévères et résistantes à travers le monde doit inciter à multiplier et renforcer les mesures prophylactiques.

## Introduction

Décrite la première fois au Maroc en 1914 par Foley et Vialate [1], la leishmaniose cutanée (LC) constitue un problème de santé publique dans notre pays. Elle est en nette recrudescence, due à un protozoaire flagellé appartenant au genre leishmania. Le but de notre travail se propose de décrire les caractéristiques épidémiologiques, cliniques et évolutives de la LC dans notre expérience.

## Méthodes

A travers une étude rétrospective, observationnelle menée au service de dermatologie de l'hôpital militaire d'instruction Mohamed V de Rabat, durant la période de janvier 2003 à décembre 2012, incluant les patients ayant le diagnostic de leishmaniose cutanée avec confirmation parasitologique et histologique. Pour chaque patient ont été précisés: l’âge, le sexe, l'origine géographique, la durée d’évolution, l'aspect clinique, le schéma thérapeutique utilisé ainsi que l’évolution sous traitement. L'analyse statistique a été effectuée en utilisant le logiciel SPSS (version 17).

## Résultats

**Figure 1:**
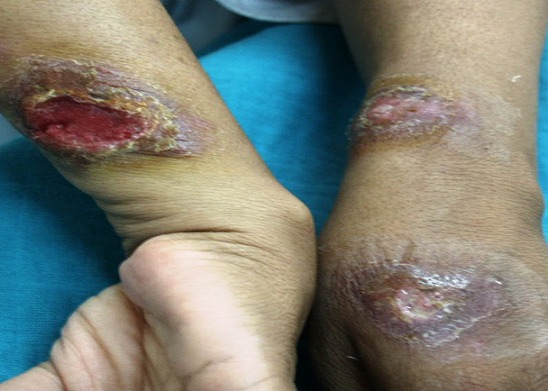
Leishmaniose ulcéro-croûteuse.

**Figure 2:**
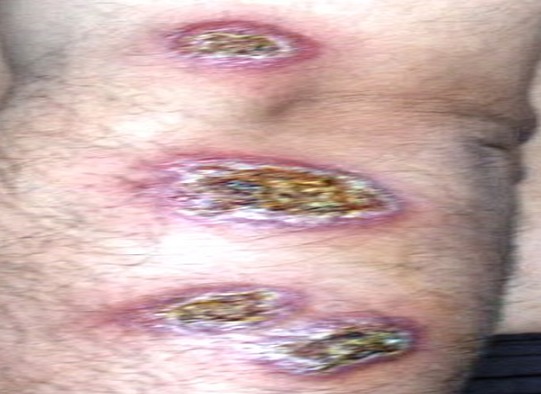
Leishmaniose sporotrichoide

**Figure 3:**
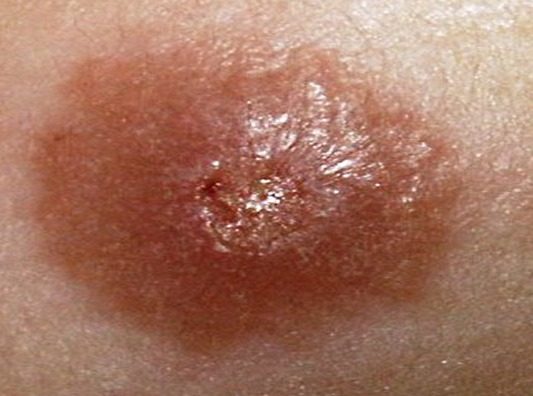
Leishmaniose papulo-nodulaire

Sur la période étudiée, 157 patients ont été colligés. L’âge de nos patients variait de 6 à 63 ans avec une moyenne de 34.5 ans. Une nette prédominance masculine a été notée (70%). Les patients étaient originaires du sud du Maroc dans 59% des cas, du centre dans 30% et du nord du pays dans 11% des cas. Le délai moyen de consultation était de 3.6 mois (2 mois-10 mois). Les lésions étaient multiples dans 48,5% avec une moyenne de 3.25 lésions par malade. Les lésions siégeaient sur la face dans 29.6% des cas, sur les membres supérieurs dans 44.4%, sur les membres inferieurs dans 18.5% et sur le tronc dans 7.4% des cas. Les aspects cliniques retrouvés (Figure 1, Figure 2, Figure 3) étaient: la forme ulcéro-croûteuse 48%, la forme papulo-nodulaire 34%, la forme érythémato-squameuse 11% et la forme sporotichoide 7%.

**Figure 4:**
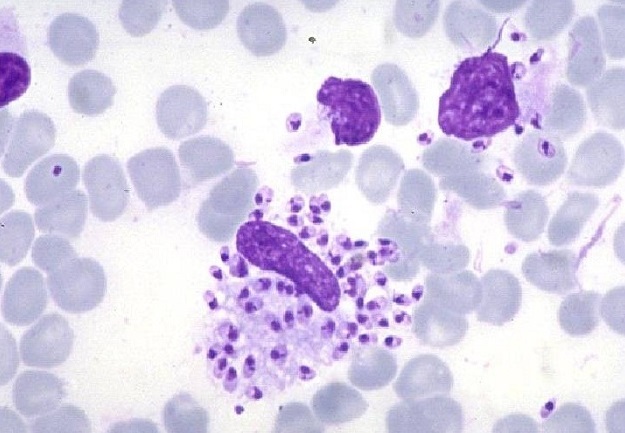
Examen parasitologique objectivant des corps de Leishman en intramacrophagique à l'examen direct

**Figure 5:**
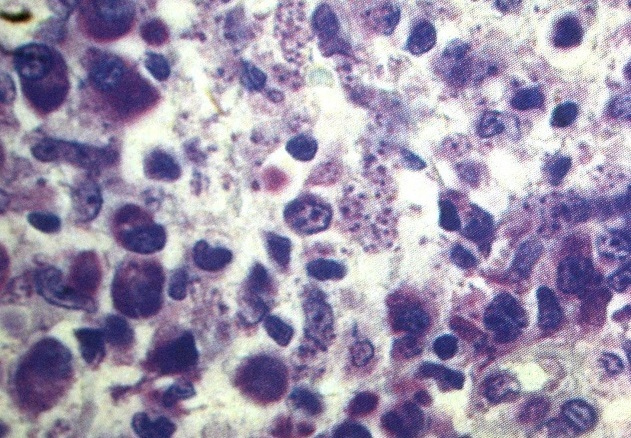
Les corps de Leishman objectivées après coloration PAS (Periodic acid schiff)

L'examen parasitologique a été réalisé chez tous les patients et il a été positif chez 75% (Figure 4). L’étude histologique a été réalisée chez tous les patients, et avait objectivée un infiltrat dermique de type granulomateux caractérisé par la présence simultanée de plasmocytes et de cellules épithelioides avec dans certains cas des microfoyers de nécrose fibrinoide, la coloration de MGG a permis de mettre en évidence des corps de leishman dans 55% des cas (Figure 5). Le Glucantime était utilisé dans 29.3% des cas, en intralésionnelle chez 31patients et par voie générale chez 15 patients, l'azote liquide était utilisé chez 111 autres. L’évolution post-thérapeutique était favorable avec disparition quasi-complète des lésions dans un délai variant de 6 à 10 semaines chez les patients qui avaient reçus le Glucantime, au prix de cicatrices inesthétique chez 14 patients. La guérison était obtenue dans 76% des cas après 3 mois de traitement chez les patients qui avaient reçus l'azote liquide et au bout de quatre à six séances.

## Discussion

Les leishmanioses cutanées possèdent une aire géographique circumterrestre. Selon l'OMS, la population exposée au risque de leishmaniose est estimée à 350 millions de personnes [1]. L'incidence mondiale des LC, toutes formes cliniques confondues, est comprise entre 1 et 1,5 millions/an. Plus de 90% des cas de LC sont issus de l'Afghanistan, de l'Iran, de l'Arabie saoudite et de la Syrie pour l'ancien monde, du Brésil et du Pérou pour le nouveau monde [2].

Le Maroc constitue un pays d'endémie de la LC. L'infection sévit sous trois formes noso-géographiques: la LC zoonotique à *Leishmania major* au sud, la LC anthroponotique à *Leishmania tropica* au centre avec émergence de nouveaux foyers au nord et la LC sporadique à *Leishmania infantum* au nord dont le premier cas marocain a été révélé en 1996 [1, 3, 4].

Entre 2007 et 2011, 27457 cas de LC ont été recensés [5]. L'analyse des données épidémiologiques a dévoilée une maîtrise du profil de la LC à *L. major* dans la majorité des anciens foyers avec une réactivation constatée au cours de l'année 2010. Par ailleurs, la LC à *L. tropica* a connu des poussées épidémiques.

Trois types de foyers ont été déterminés: foyers de faible endémicité, de moyenne endémicité et de forte endémicité, circonscrits dans les provinces du centre et du nord.

Sur le plan clinique, nos résultats sont superposables à ceux de la littérature. La prédominance de la forme ulcéro-croûteuse dans notre série, notée également dans les autres études effectuées en Afrique du nord, est expliquée par la fréquence de la LC à L. Major [3]. Cette dernière forme se caractérise par des lésions volontiers multiples, la localisation au niveau des régions découvertes surtout au niveau des membres ainsi que la courte durée d’évolution [6].

Le délai long de consultation retrouvé dans notre travail s'explique par le caractère lentement progressif et indolent des lésions. Dans notre contexte, le diagnostic est souvent fait en automne ou en hiver à distance de la contamination qui a eu lieu en été (après un séjour en zone d'endémie).

De nombreuses présentations cliniques sont possibles au cours de LC, des formes impétigoides, verruqueuses, végétantes, lupoides, pseudotumorales, psoriasiformes, lichénifiées, ulcéreuses, echtymateuses, lymphangitiques, abortives, sporotrichoides et nodulaires. Ce polymorphisme clinique ne dépend pas uniquement des caractères génétiques du parasite, mais aussi du statut immunologique de l'hôte. En effet, la balance des phénotypes fonctionnels des lymphocytes T CD4 joue un rôle important dans le déterminisme de cette affection; schématiquement la réponse de type Th1 correspond à une lésion localisée bénigne, la réponse Th2 implique une lésion sévère extensive.

La coinfection leishmania-VIH est considérée actuellement comme une maladie émergente surtout en Europe méridionale [7]. L'OMS estime que 1.5 à 9% des malades ayant un sida sont atteints d'une leishmaniose viscérale [2]. La forme cutanée est de plus en plus décrite au cours de l'infection VIH [8].

Le diagnostic de LC est évoque cliniquement, la confirmation repose sur la mise en évidence du parasite. L'examen direct sur le frottis ou la ponction colorée au MGG semble être le meilleur examen pour le diagnostic car économique, facile, rapide et sans danger. Cependant, il manque de sensibilité. La mise en culture sur milieu spécial(NNN) permet l'amélioration de 16% de la sensibilité de l'examen parasitologique [9, 10]. L'examen anatomopathologique contribue également au diagnostic. L'identification enzymatique d'espèce est une technique de référence, réservée aux laboratoires spécialisés, de même que pour la PCR dont les résultats semblent très prometteurs (98% de sensibilité versus 80% avec les moyens de diagnostic classiques) [11, 12]. Ceci fait discuter l'intérêt de cette nouvelle méthode dans notre contexte devant une présentation clinique évocatrice sans isolement du parasite aux techniques usuelles. Dans notre expérience d'autres arguments ont été pris en compte: la notion de séjour en zone d'endémie, l’évolution lente et la non réponse aux autres thérapeutiques (antibiotiques, antimycosiques).

La thérapeutique des leishmanioses n'a connu que des changements limités depuis de nombreuses années. Le Glucantime constitue le traitement de référence des leishmanioses cutanées [13]. Cependant ce traitement expose à de nombreux effets secondaires, des résistances sont de plus en plus rapportées dans plusieurs pays. Pour les LC uniques ou peu nombreuses, l'infiltration intra-lésionnelle a prouvée son efficacité (100% de succès dans notre série). La voie générale est réservée aux formes diffuses. L'intérêt de la cryothérapie est rapporté dans différentes études avec une efficacité avoisinant 84% au bout de quatre séances. Les résultats obtenus chez nos patients rejoignent ceux de la littérature, avec une efficacité de la cryothérapie presque égale au Glucantime sans effets indésirables généraux ni cicatrices pigmentées. De nombreux produits ont été essayés dans la littérature (l'amphotéricine B, le fluconazole, la disulone, la rifampicine), ainsi que le laser, la bléomycine en intra-lésionnel. Actuellement, des espoirs sont placés dans l'allopurinol, l'aminosidine sulfate (la paromomycine) ou les triazolés, voire dans certaines hydroxynaphtoquinones, telle l'atovaquone. L'immunostimulation par interféron a fait l'objet d'essais cliniques probants malheureusement sans lendemain [13, 14].

## Conclusion

La leishmaniose cutanée continue à poser un vrai problème de santé publique dans notre pays. L’émergence de formes sévères et résistantes à travers le monde doit inciter à multiplier et renforcer les mesures prophylactiques à travers la lutte contre les réservoirs et les vecteurs du parasite. Une optimisation thérapeutique, par le suivi de protocoles standardisés et par un référentiel consensuel reste nécessaire.
